# Advance care planning before and during the COVID-19 pandemic: an observational cohort study of 73 675 patients’ records

**DOI:** 10.3399/BJGPO.2023.0145

**Published:** 2024-10-16

**Authors:** Philippa G McFarlane, Catey Bunce, Katherine E Sleeman, Martina Orlovic, Jonathan Koffman, John Rosling, Alastair Bearne, Margaret Powell, Julia Riley, Joanne Droney

**Affiliations:** 1 The Royal Marsden NHS Foundation Trust, London, UK; 2 The Cicely Saunders Institute, King’s College London, London, UK; 3 Wolfson Palliative Care Research Centre, Hull York Medical School, Heslington, UK; 4 Institute of Global Health Innovation, Imperial College London, London, UK

**Keywords:** advance care planning, primary health care, terminal illness, palliative care

## Abstract

**Background:**

Advance care planning (ACP) was encouraged by policymakers throughout the COVID-19 pandemic. Little is known about use of ACP during this time.

**Aim:**

To compare use of ACP before and during the COVID-19 pandemic.

**Design & setting:**

Retrospective, observational cohort study comparing the creation, use, and content of Electronic Palliative Care Coordination System (EPaCCS) records in London. Individuals aged ≥18 years with a Coordinate My Care record, created and published in the pre-pandemic period (1 January 2018–31 December 2019), wave 1 (W1; 20 March 2020–4 July 2020), interwave (IW; 5 July 2020–30 September 2020), and wave 2 (W2; 1 October 2020–5 March 2021).

**Method:**

Patient demographics and components of ACP were compared using descriptive and comparative statistics.

**Results:**

In total, 73 675 records were included; 35 108 pre-pandemic, 21 235 W1, 6323 IW, and 9925 W2 (*n* = 1084 records not stratified as created and published in different periods). Most records were created in primary care (55.6% pre-pandemic, 75.5% W1, and 47.7% W2). Compared with the pre-pandemic period, the average weekly number of records created increased by 296.9% W1 (*P*<0.005), 35.1% IW, and 29.1% W2 (*P*<0.005). Patients with records created during the pandemic were younger (60.8% aged ≥80 years W1, 57.5% IW, 59.3% W2, 64.9% pre-pandemic [*P*<0.005]). Patients with records created in W1 had longer estimated prognoses at record creation (73.3% had an estimated prognosis of ≥1 year W1 versus 53.3% pre-pandemic [*P*<0.005]) and were more likely to be 'for resuscitation' (38.2% W1 versus 29.8% pre-pandemic [*P*<0.005]).

**Conclusion:**

During the COVID-19 pandemic increased ACP activity was observed, especially in primary care, for younger people and those not imminently dying. Further research is needed to identify training and planning requirements as well as organisational and system changes to support sustained high-quality ACP within primary care.

## How this fits in

Advance care planning (ACP) enables individuals to define goals and preferences for future medical care in collaboration with family members and healthcare professionals. It is regarded as an essential component of high-quality end-of-life care. The digital sharing of ACP records facilitates enhanced care coordination across healthcare settings. Both GPs and patients became more aware and open to discussing ACP during the COVID-19 pandemic; however, large scale analysis of ACP activity in the primary care setting during the pandemic is lacking. Evaluation of digital advance care plans demonstrates a significant increase in activity at the start of the pandemic, with an increase in the proportion of records created in primary care and with more individuals who were not imminently dying engaging with ACP during the first wave. Further work is needed to understand the levels of influence (personal, inter-personal, provider, and societal) underpinning this behaviour change and how these factors relate to clinical practice in the post-pandemic era.

## Introduction

Advance care planning (ACP) enables *'individuals to define goals and preferences for future medical treatment and care, to discuss these goals and preferences with family and healthcare providers, and to record and review these preferences'*.^1^ ACP is a dynamic component of high-quality end-of-life care, which is endorsed in policy internationally.^2–5^ ACP facilitates treatment and care that aligns with the individual’s preferences and ideally reflects changes in wishes and goals over time.^6^ To be meaningful in informing patient-centred clinical care, ACP records need to be accessible to healthcare professionals across different settings (primary care, emergency services, accident and emergency departments, hospitals, community nursing teams, hospices, and care homes). Electronic Palliative Care Coordination Systems (EPaCCS) are increasingly used in the UK to digitally share person-centred health data and ACP records, and enable care coordination across many healthcare settings with demonstrated use in primary care.^7,8^

Since the start of the COVID-19 pandemic, morbidity and mortality forecasts highlighted the need to prepare people considered to be at risk for potentially fatal illness, especially people who were frail or had other serious conditions.^9,10^ ACP was encouraged to facilitate individualised and holistic patient-centred care for these individuals.^11–14^ Internationally, guidelines were developed to support ACP,^15,16^ with calls for support from the primary care sector to facilitate these conversations.^17^ Together with extensive media coverage, increased awareness, and discussion about death and dying, higher engagement in ACP resulted.^18–20^ Yet, particularly across the primary care setting, there were also fears of inappropriate, population-based decision making occurring, with 'blanket' Do Not Attempt Cardiopulmonary Resuscitation (DNACPR) recommendations being made without individualised considerations.^21–24^

Engagement with ACP at an individual patient level during the pandemic remains poorly understood. Examination of data contained within EPaCCS offers a unique lens to focus down on ACP during this period, and across heath settings, to inform future practice and policy. This study aimed to examine changes in ACP engagement during the pandemic through a comparison of the creation, use, and content of London-wide EPaCCS records before and during the onset of COVID-19.

## Method

### Study design

This was a population-based, retrospective, observational cohort study of anonymised data collected within routine clinical practice.

### Population

EPaCCS records were created and published on the Coordinate My Care system (https://www.coordinatemycare.co.uk) between 1 January 2018 and 5 March 2021 for individuals aged ≥18 years who had consented for their anonymised data to be used for research purposes when creating a record.

### Setting

Coordinate My Care is a digital clinical service developed to support urgent and ACP for patients with complex and life-limiting conditions. It is the largest EPaCCS in England serving 8.9 million people across London between 2010 and 2022.^25^ Coordinate My Care records are created through collaboration between healthcare professionals and patients and families. Published plans can be accessed and updated in real-time by patients, primary, secondary, and community care services, as well as by urgent care providers. Records contain clinical, demographic information, and patients’ wishes and preferences for care, which can be accessed by healthcare providers in urgent situations or if the individual loses mental capacity to engage in health-related decision making.

### Patient and public involvement

In keeping with UK standards for patient and public involvement (https://sites.google.com/nihr.ac.uk/pi-standards/home), three dedicated 'experts by experience' were actively involved throughout this study.

### Data analysis

Analyses were stratified according to the major surges or 'waves' during the first year of the pandemic. Dates were aligned with the timings of the government restrictions and public health data regarding deaths in England and London, specifically:

1. pre-pandemic period, between 1 January 2018 and 31 December 2019 (104 weeks);
2. wave 1 (W1) of the pandemic, between 20 March 2020 and 4 July 2020 (15 weeks);
3. wave 2 (W2) of the pandemic, between 1 October 2020 and 5 March 2021 (22 weeks); and
4. the inter-wave (IW) period, the time between these two waves (5 July 2020–30 September 2020 [12.5 weeks]).

The pre- and post-pandemic cohorts were described using counts and proportions. Variables within the W1, IW, and W2 cohorts were statistically compared with the pre-pandemic cohort using a two-way proportional z-test in terms of demographics, creation, content, and use of Coordinate My Care records. The number of records created per week, the patient characteristics, and the setting of the initial record creation were explored. Content of the advance care plans were examined in terms of documented decisions about cardiopulmonary resuscitation, the ceiling of treatment, and end-of-life-care preferences. The number of times that records were accessed by professionals working in urgent (ambulance and emergency call services [NHS 111, 999]) and non-urgent healthcare settings was assessed. No correction has been made for multiple testing within these exploratory analyses. Data were analysed using Stata (version 16) software. Data were reported according to the REporting of studies Conducted using Observational Routinely-collected health Data (RECORD) guidelines.^26^

### Regulatory and ethics committee approval

In accordance with the guidance from the NHS Health Research Authority (HRA) Medical Research Council UK, the research was exempt from NHS Research Ethics Committee review as it involved the secondary use of anonymised data collected in the course of normal care.^27^

## Results

### ACP record creation

**Figure 1.**
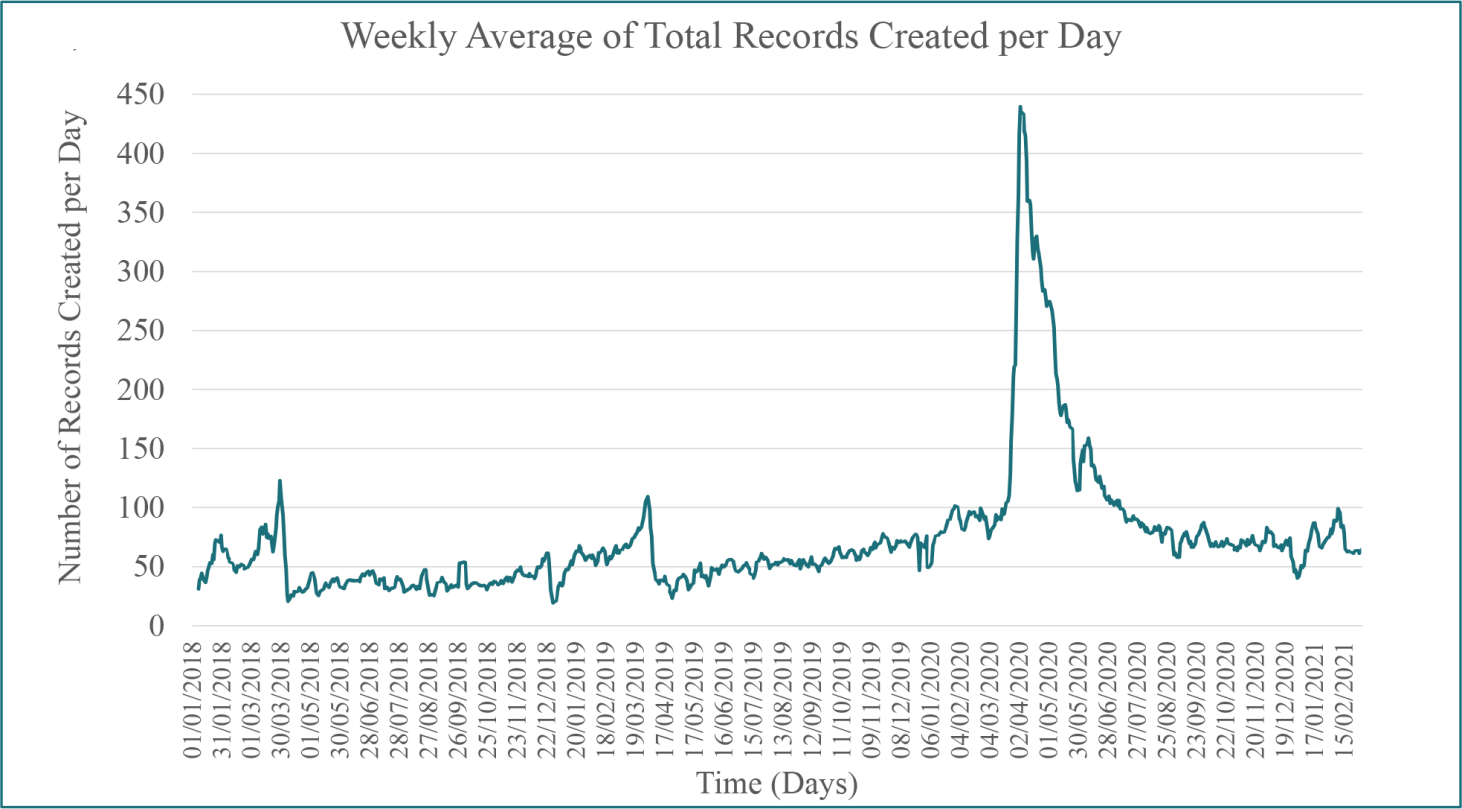
Weekly average total number of records created per day between 1 January 2018 and 5 March 2021

**Figure 2.**
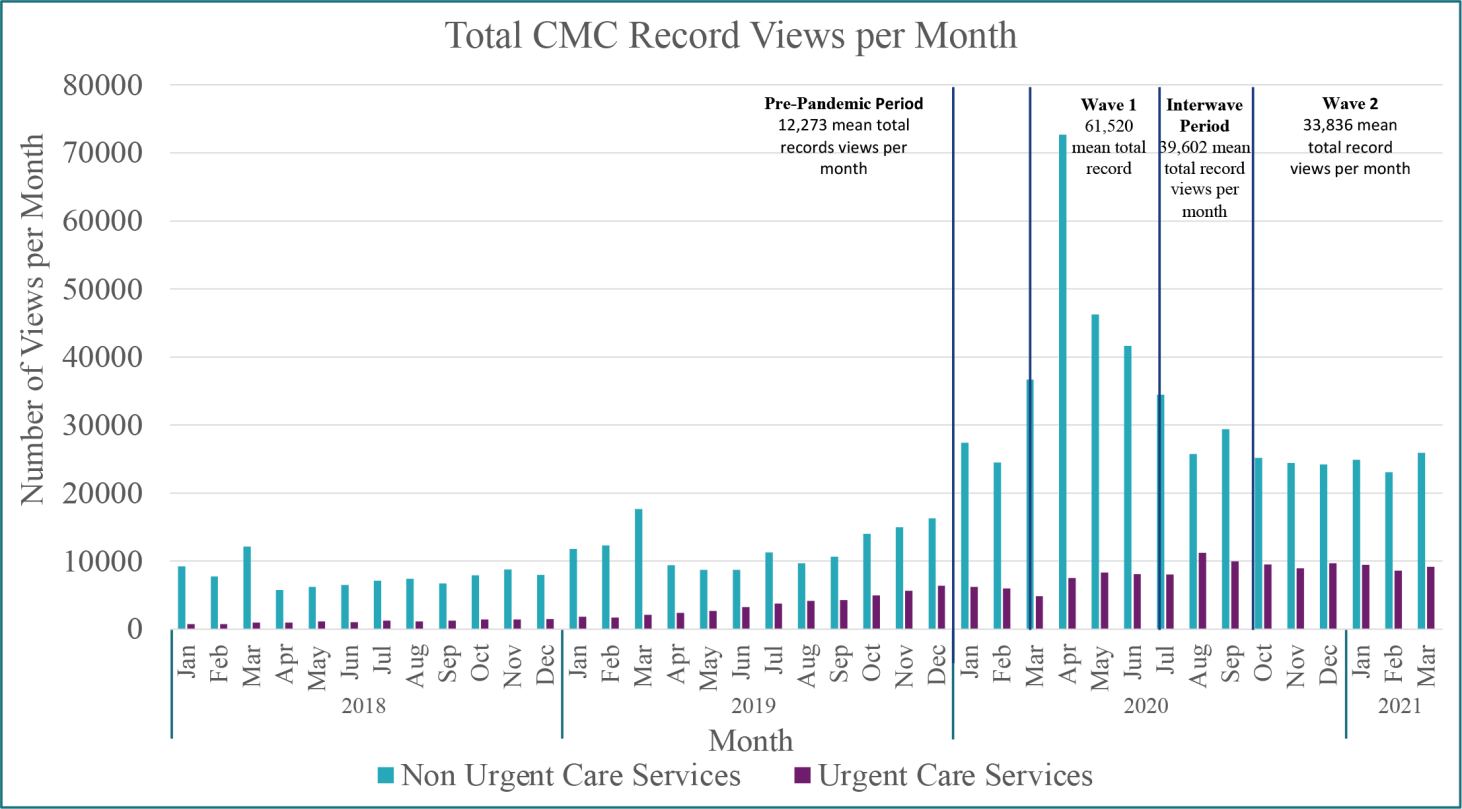
Monthly total Coordinate My Care record views for non-urgent and urgent care services (NHS 111 and 999). CMC = Coordinate My Care.

In total, 73 675 records were included in the analysis, of which 35 108 were in the pre-pandemic cohort and 38 567 records in the pandemic cohort (*n* = 21 235 W1, *n* = 6323 IW, and *n* = 9925 W2 [*n* = 1084 records were not statified by wave due to records being created and later published in different time periods: *n* = 728 W1 and *n* = 356 IW). Compared with the pre-pandemic period, the average number of records made per week (*n* = 338 pre-pandemic) increased by 296.9% in W1 (*P*<0.005) from 357 per week to 1416 per week, 35.1% in the IW period, and 29.1% in W2 (*P*<0.005) (Figures 1 and 2).

Compared with the pre-pandemic period, individuals who created records during the pandemic were younger (60.8% aged ≥80 years W1, 57.5% IW, 59.3% W2, and 64.9% pre-pandemic [*P*<0.005]) (see Supplementary Table S1). Individuals who created records in W1 had a better performance status, and in both W1 and the IW period individuals had a longer estimated prognoses than those in the pre-pandemic cohort (73.3% had an estimated prognosis of ≥1 year W1 versus 53.3% pre-pandemic [*P*<0.005]). In W1, there was a decrease in the proportion of patients who had a primary diagnosis of cancer, and a reciprocal rise in the proportion of respiratory patients and the ‘other’ diagnosis group, which included endocrine, haematological, mental health, musculoskeletal, vascular, and ‘other’ disorders, was seen. More individuals were living in care homes in W1 and IW periods, but by W2 fewer people who created records were living in care homes. More records were made by individuals who could not consent to create a Coordinate My Care record throughout the pandemic. For these individuals, a Coordinate My Care record was created in the individual's ‘best interests’ or with the involvement of a ‘health and welfare lasting power attorney’. Fewer individuals who created records in W1 were living in areas of highest deprivation (13.2% W1 versus 15.7% pre-pandemic).

Both before and during the COVID-19 pandemic, the highest proportion of records were created in the primary care setting (see Supplementary Table S1). In W1, 75.5% (*n* = 16 030) of all records were created in primary care, compared with 55.6% (*n* = 19 529) at baseline. This increase was not sustained, and proportions fell to just under pre-pandemic levels by W2 (*n* = 4733, 47.7%) in line with a higher proportion being created in acute trusts.

### Use of ACP records

Throughout the pandemic there was a marked increase in the number of times that these records were viewed each month in both non-urgent and urgent settings (Figures 1 and 2 and Supplementary Table S2). Compared with the pre-pandemic period, the mean monthly non-urgent (*n* = 9956) and urgent record (*n* = 2317) views, respectively, increased by 437.6% (*n* = 53 525 mean views) and 245.1% (*n* = 7995 mean views) in W1, by 200.1% (*n* = 29 874 mean views) and 319.8% (*n* = 9727 mean views) in IW, and by 147.1% (*n* = 24 600 mean views) and 298.6% (*n* = 9236 mean views) in W2.

### Content of ACP records: engagement with ACP

Compared with the pre-pandemic period, fewer records created in W1 had DNACPR recommendations recorded (61.8% in W1 versus 70.3% pre-pandemic*, P*<0.005) but this increased to pre-pandemic levels by W2 to 72.7% (see Supplementary Table S3). A higher proportion of individuals in W1 (32.6% versus 25.7% pre-pandemic) had full-active medical intervention recorded as their ceiling of treatment and more individuals wanted to be in a care home (26.6% versus 24.3% pre-pandemic) or hospital (13.3% versus 5.8% pre-pandemic) setting. By W2 these changes reverted to pre-pandemic levels (19.8%, 20.6%, and 5.7%, respectively).

## Discussion

### Summary

To the authors’ knowledge, this is the first study to examine the creation, use, and content of advance care plans during the pandemic using a large established EPaCCS. Although increased engagement with ACP at this time has been reported by healthcare staff,^19,28^ this is the first study, to the authors’ knowledge, to quantify that increased activity on a large scale, across multiple aspects of the health service and a wide urban geographical area.

Previous research has highlighted the central role of primary care in ACP discussions.^29^ Before the pandemic, uptake of ACP was variably reported, with 25%–65% of patients at the end of life having an element of ACP within primary care.^30–32^ In our study, 55% of all pre-pandemic records were created in primary care, clearly illustrating the central role of primary care in the creation of advance care plans. Our data additionally demonstrate the large increase in engagement with ACP within primary care during the first wave of the pandemic, reaching 75%.

During the first wave of the pandemic, 1416 records were created each week, across all healthcare settings, compared with 338 per week in the pre-pandemic period, demonstrating a significant increase in the number of digitally supported advance care plans. This increased activity was sustained but at a lower level throughout the initial year of the pandemic. Individuals who made advance care plans early in the pandemic were younger, had better performance status, longer prognoses, and were more likely to have a preference to be cared for and die in a hospital, compared with pre-pandemic. In the US, individuals from ethnically diverse and minoritised communities and those from lower socioeconomic positions are disadvantaged in terms of accessing and engaging with ACP.^33–36^ Apart from a small decrease in individuals living in areas of highest deprivation in W1, our data did not show significant differences in engagement with ACP across the COVID-19 period according to socioeconomic status.

Amid concerns regarding the influence of rationing on clinical decision making in end-of-life care,^19,21–24,37^ the importance of ACP and appropriate decision making, particularly regarding DNACPR recommendations, has been highlighted.^13,28^ Although we cannot determine rates of DNACPR recommendations at population level, in this dataset the proportions of total patients with a documented DNACPR decision fell during the initial months of the pandemic.

### Strengths and limitations

This novel study examining the engagement with ACP during the first year of the pandemic, via the lens of the largest known EPaCCS, provides a comprehensive insight into engagement with the components of ACP in a large metropolitan area. The large cohort sizes across the three main timeframes of the pandemic’s first year have additionally allowed for a more refined exploration of engagement patterns, granting particular focus into the ACP activity in primary care. Our data has additionally facilitated important insights into resuscitation decisions during a period of concerns regarding these decisions.^21–23^

Our study has several limitations. As we cannot determine from our data why ACP activity increased, we have drawn on relevant literature to propose several hypotheses to explain this. Qualitative research among individuals living with life-limiting conditions, their families, and healthcare professionals would provide insight into the experience of ACP, the degree of shared decision making, and the use of electronic ACP record systems during this time. Owing to significant missing data, some key factors such as ethnicity, formal care, and family support were precluded from our analysis, limiting our ability to explore social determinants of ACP engagement. The findings from this study set in London may not be generalisable to other settings with different access to healthcare providers. The data were extracted from Coordinate My Care, an EPaCCS, which was established within routine care before the pandemic; variations in the availability of and level of interoperability of EPaCCS in other areas also impact the generalisability of our findings.

### Comparison with existing literature

Evaluation within a socio-ecological framework has identified multiple interacting 'levels of influence' underpinning the uptake of, and engagement with, ACP,^38,39^ including during COVID-19.^19^ When applied to ACP in primary care, these include factors relating to the individual patient themselves (knowledge about ACP and perceived relevance depending on individual health status), interpersonal and relationship factors between the patient and others (including family and healthcare professionals), factors at the level of healthcare teams and services (based largely on knowledge, competence, and time), and systems-level factors (including culture, funding, electronic systems, and policy).^40^ Individual-level factors specific to COVID-19 included complex decision making in the face of a new and, at that time, a poorly understood disease, while interpersonal factors highlighted the communication challenges posed by social distancing and wearing personal protective equipment.^41^

In non-COVID-19 times, 80% of individuals acknowledged the benefits of ACP; however, 67% did not wish to think about it.^40,42^ The considerable increase in ACP engagement demonstrated during the first wave of the pandemic might reflect raised public awareness,^43^ framed within a global *'context of fear and uncertainty'*, resulting in *'upstreamed'* and *'normalised'* ACP discussions.^44^ As seen in wartime, over time, the perceived threat and emotions relating to the pandemic have stabilised.^45^ We hypothesise that this adaptation may have lessened some of the immediate pandemic-related motivators to engage with ACP, resulting in the relative fall in engagement after the surge in W1.

At a provider level, workload pressures have been cited as barriers to ACP both before and during COVID-19, while at a systems level, there have been issues reported about how to share ACP information across multiple settings, including primary care.^19,46^ Our data reflects the considerable workload undertaken in primary care to enable and facilitate ACP throughout the pandemic, especially during the initial months. Interview data during COVID-19 highlights the role GPs played in providing information about and facilitating ACP.^41^ In our study, engagement with ACP at such a scale may have been facilitated by the already established London-wide EPaCCS, Coordinate My Care, with pre-existing links between many different healthcare providers.^47^ Further work is needed, however, to better understand the impact of established technological infrastructures in facilitating ACP engagement.

### Implications for research and practice

As highlighted by the significant increase in record creation, our data show that patients and clinicians were willing to engage with ACP, even during a global crisis. To promote and enhance sustained uptake and engagement with ACP, consideration must be given to the above-mentioned levels of influence. Uptake may be improved through proactive identification of opportunities for introducing ACP discussions ahead of clinical deterioration.^46,48^ This supports the dual framework of living as well as possible while simultaneously recognising the possibility of deterioration and death.^49^ Such measures may 'normalise' ACP within routine clinical care. Identification of patients who may benefit from proactive ACP, including those with non-cancer diagnoses and care home residents, is an area that requires ongoing development and evaluation.^32,43,50^

Studies in primary healthcare services in the UK and the Netherlands report increased ACP initiation and provision during the pandemic.^41,51^ With the high proportion of records created in the primary care setting, our data also highlight the invaluable role of primary care in supporting patients to engage with ACP. The normalisation of ACP within primary care (and other areas of the health service) requires training and competency planning, as well as an organisational and systems approach to promote and support high-quality ACP.

The demographics of people creating records in W1 in our data demonstrate that individuals not imminently dying can also engage with ACP and create ‘living plans’. These documented preferences and priorities support ACP as a process rather than a one-off discussion^52^ and also hold significance for influencing policy.

Evaluation of outcomes of patients with EPaCCS records before and during the COVID-19 pandemic demonstrates the potential for digital records to personalise care and influence end-of-life experiences.^53^ Lacking information coordination between healthcare professionals is a recognised barrier to engagement with ACP,^46^ emphasising the importance of suitable digital solutions to underpin the documentation, updating, and sharing of ACP discussions during the pandemic.^43^ With death and dying increasingly normalised since the pandemic’s onset, there is an opportunity for leadership, education, and enhanced use of digital health solutions to overcome barriers to ACP as we face the para-pandemic and beyond.^42,43^

In conclusion, our data highlight the role of primary care in supporting patients to engage with ACP during the pandemic, further evidencing the important role GPs have in the coordination and future planning of care for patients with advanced illness. The normalisation of ACP within primary care (and other areas of the health service) requires continued training and competency planning, as well as an organisational and systems approach to promote and support sustained high-quality ACP and evaluation of impact on patient outcomes. The pandemic has additionally emphasised the importance of suitable digital solutions to underpin the documentation, updating, and sharing of ACP discussions. As the world moves into the post-pandemic era, learning must be applied to ongoing patient care and systems to support ACP facilitation and engagement.

## Data

Anonymised data from this study will be stored on the Biomedical Research Informatics Digital Environment (BRIDgE), a Trusted Research Environment and informatics platform at The Royal Marsden Biomedical Research Centre. Data sharing requests and access to the protocol and supplementary information would be available on reasonable request after completion of existing studies and whenever legally and ethically possible. Data access requests should be directed to Joanne Droney (joanne.droney@rmh.nhs.uk). Once approved, projects intending to use the data will be reviewed and authorised by The Royal Marsden Committee for Clinical Research and access provided via the Trusted Research Environment.
